# A pilot study of 4′-[methyl-^11^C]-thiothymidine PET/CT for detection of regional lymph node metastasis in non-small cell lung cancer

**DOI:** 10.1186/2191-219X-4-10

**Published:** 2014-03-05

**Authors:** Ryogo Minamimoto, Jun Toyohara, Hideyuki Ito, Ayako Seike, Yoko Miyata, Miyako Morooka, Momoko Okasaki, Kazuhiko Nakajima, Kimiteru Ito, Kiichi Ishiwata, Kazuo Kubota

**Affiliations:** 1 Division of Nuclear Medicine, Department of Radiology, National Center for Global Health and Medicine, 1-21-1, Toyama, Shinjyuku-ku, Tokyo 162-8655, Japan; 2 Research Team for Neuroimaging, Tokyo Metropolitan Institute of Gerontology, Tokyo, Japan; 3 Department of Thoracic Surgery, National Center for Global Health and Medicine, Tokyo, Japan

**Keywords:** 4DST, FDG-PET/CT, Lymph node metastasis, Non-small cell lung cancer (NSCLC), Cell proliferation

## Abstract

**Background:**

4′-[methyl-^11^C]-thiothymidine (4DST) is a novel positron emission tomography (PET) tracer to assess proliferation of malignancy. The diagnostic abilities of 4DST and 2-deoxy-2-^18^ F-fluoro-d-glucose (FDG) for detecting regional lymph node (LN) metastases of non-small cell lung cancer (NSCLC) were prospectively compared. In addition, the relationship between the PET result and the patient's prognosis was evaluated.

**Methods:**

A total of 31 patients with NSCLC underwent 4DST PET/computed tomography (CT) and FDG PET/CT. The PET/CT images were evaluated qualitatively and quantitatively for focal uptake of each PET tracer, according to the staging system of the American Joint Committee on Cancer. Surgical and histological results provided the reference standards. Patients were followed for up to two years to assess disease-free survival.

**Results:**

On a per-lesion basis, sensitivity, specificity, positive predictive value, negative predictive value, and accuracy for LN staging were 82%, 72%, 32%, 96%, and 73%, respectively, for 4DST, and 29%, 86%, 25%, 88%, and 78%, respectively, for FDG. The sensitivity of 4DST was significantly higher than that of FDG (*P* < 0.001). The disease-free survival rate with positive 4DST uptake in nodal lesions was 0.35, which was considerably lower than the rate of 0.83 with negative findings (*P* = 0.04). Among the factors tested, nodal staging by 4DST was the most influential prognostic factor (*P* = 0.05) in predicting the presence of a previously existing spread lesion or of a recurrence over the course of 2 years.

**Conclusion:**

4DST PET/CT is sensitive for detecting mediastinal lymph node metastasis in NSCLC, but its low specificity is a limitation. However, it may be helpful in predicting the prognosis of NSCLC.

## Background

Lymph node (LN) involvement and distant metastasis of non-small cell lung cancer (NSCLC) are indicators of a poor prognosis [1]. The 5-year survival has been shown to decrease with the extent of LN involvement in cases with any T designation (any T) and without extra-nodal metastatic disease (M0) [2]. Clinical staging can sometimes indicate the appropriate direction for therapy. Pathologic staging is still the reference standard, and the overall level of agreement between clinical staging based on thoracic computed tomography (CT) and pathological staging was only from 35% to 55% [3]. Therefore, a sensitive diagnostic tool for clinical staging has been needed to improve treatment selection in NSCLC.

Mediastinoscopy [4] and endobronchial ultrasonography-transbronchial needle aspiration (EBUS-TBNA) [5] show high sensitivity and specificity for LN staging, but they are invasive tests, and their ability to obtain a sample is dependent on the location of the lesion. Noninvasive imaging, such as CT and magnetic resonance imaging (MRI), has been used for NSCLC staging.

2-Deoxy-2-^18^F-fluoro-d-glucose (FDG) positron emission tomography (PET) has contributed to more accurate mediastinal staging of lung cancer with median sensitivity and specificity of 61% and 79%, respectively [6]. In recent studies, FDG PET/CT has been highly specific in mediastinal nodal staging (specificity 84% to 100%), but it has been sensitive in a different range (sensitivity 45.2% to 91.6%) [7-21]. Even though integrated PET/CT helps improving the accuracy of mediastinal nodal staging, it is still insufficient for detection of microscopic lymph node metastases [22].

A biologic factor intimately related to malignancy is tumor cell proliferation. Such proliferation has a prognostic relevance in various malignancies, including NSCLC [23]. Recently, a thymidine analog, carbon-11-labeled 4′-thiothymidine ([^11^C] 4DST, originally designated as [^11^C]S-dThd), was introduced as a cell proliferation imaging agent based on its mechanism of incorporation into DNA [24-26]. In our previous report, we demonstrated the great potential of 4DST-PET/CT for proliferation imaging in lung cancer itself [27]. Moreover, we showed a case which indicated the potential of 4DST for the detection of LN metastasis in NSCLC [27]. The background 4DST uptake in mediastinum was lower than that of FDG; therefore, we hypothesize that 4DST might have an advantage for detecting mediastinal lymph node metastasis. In this study, the characteristics of 4DST PET/CT for LN staging were evaluated, especially in comparison to those of FDG PET/CT. In addition, patients' prognoses according to several factors evaluated in the pre-surgical state were surveyed.

## Methods

### Patients

This prospective study was approved by the National Center for Global Health and Medicine institutional review board, and written informed consent was obtained from all patients. The inclusion criteria of this study were first histologic diagnosis of NSCLC based on bronchoscopic biopsy or cytology, clinical stage I to IIIA based on contrast-enhanced (CE) CT examination, and scheduled to complete resection of primary lung tumor without any neoadjuvant therapy. Exclusion criteria for this study were patients with age 20 years or younger, uncontrolled diabetes, pregnancy, and suggested extrathoracic metastasis by whole-body FDG-PET/CT scan; however, no patients met these exclusion criteria. A total of 31 patients (21 men and 10 women; mean (±SD) age 67.6 ± 11.7 years; range 36 to 88 years) was included consecutively (Table 1). All included patients were referred to our hospital for further testing of detected lung lesion. Most of the patients were asymptomatic and detected lung lesions by annual medical examination based on chest X-ray and/or chest CT. As a result, the high rate of early stage NSCLC was included in this study.

**Table 1:** Clinical data and PET/CT findings in 31 patients with NSCLC

**Patient no.**	**Age**	**Sex**	**Pathology**	**Tumor diameter (mm)**	**TNM classification**	**Lymph node staging**		**SUVmax**					
								**Primary lung lesion**		**Mediastinal positive uptake**		**Ascending aorta**	
						**4DST**	**FDG**	**4DST**	**FDG**	**4DST**	**FDG**	**4DST**	**FDG**
1	56	M	Adeno	31	pT2aN0M0	N0	N0	4.7	10.9	-	-	0.6	1.6
2	79	F	Adeno	14, 18	pT1aN0M0	N0	N0	1.8 to 2.1	1.3 to 2.8	-	-	1.1	2.2
3	55	F	Adeno	18	pT1aN0M0	N0	N0	4.1	7.7	-	-	1.0	1.7
4	60	M	Adeno	15	pT1aN2M0	N2	N0	1.5	1.4	1.7	-	0.7	1.7
5	69	M	Adeno	12	pT3N1M0	N2	N2	2.7	13.7	1.9 to 3.1	2.0 to 2.7	0.6	1.6
6	62	M	Adeno	32	pT2aN2M0	N2	N2	3.5	7.1	1.6 to 3.5	2.9 to 3.6	0.9	1.9
7	77	F	Adeno	20	pT1aN0M0	N0	N0	2.7	4.5	-	-	0.8	2.2
8	73	F	Adeno	15	pT2aN0M0	N3	N3	2.7	5.2	2.7 to 4.4	3.6 to 5.8	1.0	2.1
9	58	M	SCC	30	pT2aN1M0	N2	N0	3.0	5.6	2.1 to 2.5	-	0.8	1.7
10	55	M	Large cell	62	pT2bN1M0	N2	N2	3.2	7.6	1.9 to 2.0	1.7	1.0	1.8
11	72	M	Adeno	55	pT2bN0M0	N1	N0	4.5	10.1	3.0	-	0.9	2.3
12	74	M	SCC	30	pT2aN0M0	N1	N1	3.9	17.3	4.2	3.1	1.0	2.1
13	78	M	Adeno	38	pT2aN0M0	N0	N1	4.0	6.3	-	2.3	0.9	2.0
14	73	M	Adeno	20	pT1aN0M0	N2	N1	2.1	3.6	2.9 to 3.0	2.5 to 3.3	0.6	2.3
15	79	M	Adeno	19	pT1aN0M0	N2	N0	3.0	4.2	2.4 to 2.6	-	0.7	2.1
16	58	F	Adeno	18	pT1aN2M0	N2	N0	1.5	3.5	3.9 to 4.2	-	0.6	2.2
17	54	M	SCC	32	pT2aN0M0	N2	N0	1.6	0.8	2.2	-	0.7	1.6
18	84	M	Adeno	19	pT1aN0M0	N0	N0	1.9	1.2	-	-	0.5	2.1
19	76	M	Adeno	25	pT2aN0M0	N2	N0	6.5	13.5	3.0 to 5.3	-	0.6	1.8
20	75	M	Adeno	42	pT2aN2M0	N2	N0	5.2	3.6	1.7	-	0.7	2.0
21	70	M	SCC	45	pT3N1M0	N2	N0	5.6	15.7	2.8 to 2.9	-	0.4	1.7
22	73	M	Large cell	19	pT1aN0M0	N2	N0	2.8	4.2	1.7 to 2.4	-	0.6	1.8
23	76	M	Large cell	17	pT1aN2M0	N2	N2	1.7	2.8	2.1 to 3.1	4.1 to 4.4	0.4	1.5
24	88	M	SCC	70	pT3N0M0	N2	N2	5.1	18.8	4.7 to 5.6	2.7 to 3.6	0.4	1.9
25	36	F	Adeno	21	pT1bN0M0	N1	N1	4.4	3.3	2.9	2.7	0.7	2.0
26	76	F	SCC	60	pT2bN0M0	N2	N2	4.8	18.1	2.3 to 2.4	2.2 to 2.3	0.5	1.7
27	43	F	Adeno	28	pT2aN0M0	N1	N0	2.4	9.3	1.6	-	0.9	1.9
28	70	M	Adeno	20	T1bN2M1a	N2	N2	3.0	3.0	2.0 to 3.3	3.0	0.4	1.9
29	69	F	Adeno	22	T1aN0M1a	N3	N0	2.5	2.8	1.6, 1.6	-	0.6	1.7
30	70	M	Adeno	37	T2aN0M1a	N3	N0	4.7	12.2	2.6 to 4.9	-	0.7	2.3
31	61	F	Adeno	32	T2aN2M1a	N2	N2	3.3	8.9	1.7 to 4.1	3.1	0.5	1.6

NSCLC, non-small cell lung cancer; SUVmax, maximum standardized uptake value; M*,* male; F, female; Adeno, adenocarcinoma; SCC, squamous cell carcinoma; Large cell, large cell carcinoma.

A part of data from 18 of these patients was used in the previous study [27]. All patients underwent CECT before 4DST-PET/CT and FDG-PET/CT. All imaging was performed before surgical resection of the lung lesion and systematic resection of LNs. The mean interval between CE chest CT and surgery was 28 days.

### 4DST PET/CT examination

The 4DST was synthesized as previously described [27]. All subjects fasted for 5 h before receiving an intravenous injection of 4DST with a median of 716 MBq (range 283 to 777 MBq). According to our previous report [27], PET/CT images were obtained 40 min after intravenous injection of 4DST, and used either of two PET/CT systems (Biograph 16; Siemens Medical Solutions, Munich, Germany and Discovery PET/CT 600; GE Healthcare, Pewaukee, WI, USA). These systems consist of a PET scanner and a multi-detector-row CT scanner (16 detectors). Imaging covered from the vertex to the mid-thigh. Low-dose CT with shallow breathing was performed first and used for attenuation correction and image fusion. Low-dose CT data for Biograph 16 was acquired at 120 kVp using an auto exposure control system, beam pitch of 0.833, slice thickness of 5 mm, and that data for Discovery PET/CT 600 was acquired at 120 kVp using an auto exposure control system, beam pitch of 0.938, slice thickness of 3.75 mm. Emission images were acquired in three-dimensional mode for 2.5 min per bed position. PET data were reconstructed using a Gaussian filter with an ordered-subset expectation maximization algorithm (three iterations, eight subsets for the Biograph 16, and three iterations, 16 subsets for the Discovery PET/CT 600). The mean intervals between CE chest CT and 4DST-PET/CT, between 4DST-PET/CT and FDG-PET/CT, and between 4DST-PET/CT and surgery were 16, 5, and 12 days, respectively.

### FDG PET/CT examination

An in-house cyclotron and automated synthesis system (F100 or F200; Sumitomo Heavy Industries, Shinagawa, Tokyo, Japan) was used in accordance with the authorized procedure to synthesize FDG.

All subjects fasted for 5 h before blood glucose levels were measured, and the blood glucose level had to be under 120 mg/dL at the time of FDG injection. FDG was intravenously injected, and the activity was fixed at 370 MBq for 22 patients and administered at 5.0 MBq/kg of body weight (range 283 to 388 MBq) for nine patients. PET/CT images were obtained 60 min after injection with the same PET/CT machine and method as for the 4DST PET/CT examination. The mean intervals between CE chest CT and FDG PET/CT, and between surgery and FDG PET/CT were 14 and 28 days, respectively.

### PET/CT data analysis

All 4DST and FDG-PET/CT scans were evaluated in consensus of two board-certified nuclear medicine physicians blind to clinical and pathological information. Regions of interest (ROI) were placed over the lung lesion according to the CT images obtained from PET/CT and with reference to the CE chest CT image. The maximum standardized uptake value (SUVmax) was determined for 4DST and FDG PET/CT images. The size of the lung lesion was determined from the CE chest CT image.

Nodal stage was classified according to the American Joint Committee on Cancer (AJCC) staging system for the classification of lung cancer [28] in order to compare the results of PET/CT and histopathological analysis. The reference standard for the diagnosis of mediastinal metastases was surgical exploration of the mediastinum and histopathological examination of mediastinal LN compartments. Patients' TNM stages were determined according to the 7th Edition of the Lung Cancer TNM Classification; four patients who were inoperable were assessed by a combination of clinical and pathological TNM staging. Pathological diagnosis was performed by an experienced pathologist based on hematoxylin and eosin-stained tissue Sections.A positive PET scan for an N2 lesion was defined as a visually focal PET uptake that was matched to any small nodal lesions identified on the CT image of the PET/CT and with reference to the CECT image. The exact location of an N1 lesion was difficult to assess on PET/CT; thus, a PET scan was defined as positive for the N1 area if any focal uptake was confirmed in an N1 area. Any LN that existed in an N1 area was combined with that region and, by definition, generated one region named an N1 group (N1G). The SUVmax values of these visually focal uptakes were measured for both 4DST and FDG. To survey the mediastinal background uptake, an ROI was also placed on the ascending aorta as guided by the CT image from the PET/CT. With each agent, a lesion-to-background (L/B) ratio was calculated from the value of the visually focal PET uptake and the value of the background uptake.

### Patient follow-up

After resection of the primary tumor and regional LNs, any patient with a pathology-confirmed nodal metastasis received cisplatin-based or oral uracil-tegafur-based chemotherapy. Among the 27 patients who had surgical resection of the lung lesion and LN(s), 19 patients could be followed for at least 2 years for the recurrence of cancer. Of the other eight patients, two patients dropped out from this study 4 months after surgery, and the other six patients were followed-up for less than 2 years without evidence of recurrence.

The presence of a recurrent lesion was determined clinically based on the results of follow-up CE chest and abdominal CT, contrast-enhanced brain MRI, and bone scintigraphy. These tests were performed with duration between tests of 6 months or less. In the present study, disease-free survival (DFS) was defined as the absence of evidence of a previously existing spread lesion or of the recurrence of cancer over the course of 2 years after surgical procedure.

### Statistical analysis

Data are expressed as mean ± SD. Mann-Whitney's *U* test was used to compare both the SUVmax and the L/B ratio between 4DST and FDG. Sensitivity, specificity, positive predictive value (PPV), negative predictive value (NPV), and accuracy values for N staging on a per-node and a per-patient basis were calculated for 4DST and FDG.

The values are expressed as means with 95% confidence intervals (CI). The Mc Nemar chi-square test was performed to compare the sensitivity and specificity between 4DST and FDG, and the chi-square test for independence was performed to compare spread lesion or the recurrence rate between 4DST and FDG.

Univariate and multivariate logistic regression analyses were used to identify potential prognostic factors for DFS from among the following: nodal staging by 4DST, nodal staging by FDG, 4DST uptake in the lung tumor, FDG uptake in the lung tumor, lung tumor diameter, and patient age. To obtain suitable cutoff points for 4DST uptake, FDG uptake (SUVmax), and tumor diameter for primary lung cancer, receiver-operating characteristic curves were used. Two-tailed *P* values < 0.05 were considered significant.

## Results

### PET imaging and histopathological results for primary lung cancer

The first patient was enrolled on July 2010, and the last on September 2012. All primary lung tumors with histologically proven malignancy were identified by 4DST-PET/CT and FDG-PET/CT by their positive uptake. The mean SUVmax of 4DST in primary lung tumors was significantly lower than that of FDG (3.3 ± 1.3 vs. 7.2 ± 5.3; *P* < 0.002).

During surgery, four patients were deemed inoperable due to dissemination of cancer into the pleura in three patients and into the pericardium in the other. The cancer dissemination consisted of small lesions less than 2 mm in size. These small lesions had not been detected by either of the preoperative PET examinations, and they could not be seen on postoperative retrospective review. However, pleural dissemination was suspected in two of the three cases on the CE chest CT image because of slight pleural thickening and because the lung tumor was located adjacent to the pleura. The other 27 patients had surgical resection of the lung lesion with negative surgical margins and of the regional LNs. Pathologic analysis showed adenocarcinoma in 22 patients including two cases of bronchioloalveolar cancer, squamous cell carcinoma in six patients, and large cell carcinoma in three patients (Table 1).

### Detection of LN metastases

**Figure 1:**
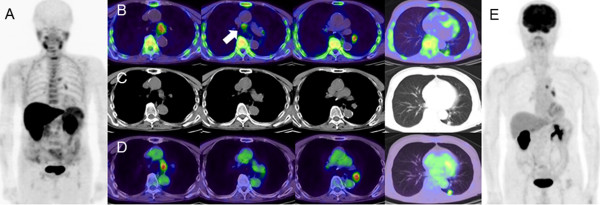
**4DST MIP (A), 4DST axial (B), CT (C), FDG axial (D), and FDG MIP images (E).** PET images (axial and maximum-intensity-projection) with 4DST and FDG for lymph node lesions and the primary lung cancer in patient No. 23. The background uptake is clearly lower in 4DST than FDG. 4DST and FDG show intense uptake at the left lower paratracheal lymph node (#4 L) and the left hilar lymph node. 4DST also shows uptake at the lower paratracheal lymph node (arrow), not clearly identified on FDG.

**Figure 2:**
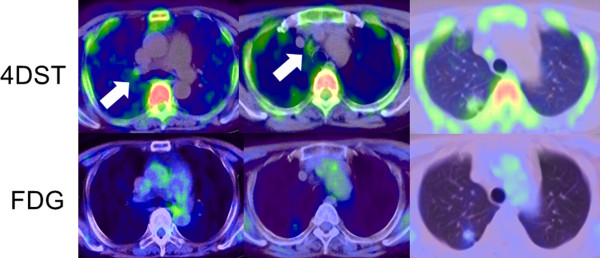
**PET findings with 4DST and FDG for lymph node lesions and the primary lung cancer in patient no. 4.** Mild but clear 4DST uptake is confirmed at the right hilar lymph node and the paratracheal nodes (#4R) (arrows).

**Figure 3:**
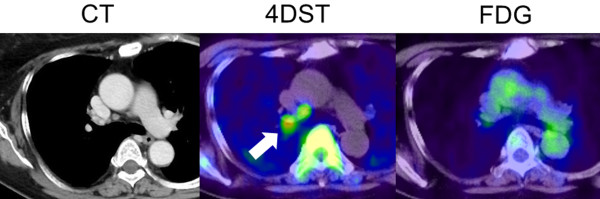
**PET findings with 4DST and FDG for lymph node lesions and primary lung cancer in patient no. 16.** Focal 4DST uptake is confirmed at the right lower paratracheal lymph node (#4R), which is not identified by FDG. The lesion was a histopathologically proven lymph node metastasis.

A total of 156 LNs (N2 area 97, N1 area 59) was resected by surgery. Of these, 21 LNs (N2 area 10, N1 area 11) proved to be positive for malignancy in nine of the 27 patients. Finally, 123 nodal groups (N2 area 97, N1G 26) were defined for 27 patients, with proven malignancy in 17 nodal groups (N2 area 10, N1G 7) of nine patients. Sensitivity, specificity, PPV, NPV, and accuracy values for N staging on a per-nodal basis and on a per-patient basis are shown in Table 2. The sensitivity on a per-node basis was significantly higher with 4DST than with FDG (82.4% vs. 29.4%; *P* < 0.002), and it was higher on a per-patient basis (66.7% vs. 22.2%), but not significantly (*P* = 0.06). In contrast, the specificity on a per-node basis was significantly lower with 4DST than with FDG (71.7% vs. 85.8%; *P* < 0.02) and also lower on a per-patient basis (33.3% vs. 61.1%), but not significantly (*P* = 0.09). 4DST PET/CT showed positive uptake for all the true positive lesions (*n* = 5) on FDG-PET/CT. However, no significant differences between 4DST and FDG were observed for PPV, NPV, or accuracy. Figures 1, 2, and 3 show the 4DST and FDG PET/CT images of patients with LN metastasis.

**Table 2:** Results of 4DST and FDG-PET/CT for N staging in 27 patients with NSCLC who underwent lymph node resection

**Subject**	**PET/CT**	**Sensitivity (%)**	**Specificity (%)**	**PPV (%)**	**NPV (%)**	**Accuracy (%)**
Per-nodal station	4DST	82.4 (0.64 to 1.00)	71.7 (0.63 to 0.80)	31.8 (0.18 to 0.46)	96.2 (0.92 to 1.00)	73.2 (0.65 to 0.81)
	FDG	29.4 (0.08 to 0.51)	85.8 (0.79 to 0.93)	25.0 (0.06 to 0.44)	88.3 (0.82 to 0.95)	78.0 (0.71 to 0.85)
Per-patient	4DST	66.7 (0.36 to 0.98)	33.3 (0.12 to 0.55)	33.3 (0.16 to 0.51)	66.7 (0.36 to 0.98)	44.4 (0.26 to 0.63)
	FDG	22.2 (−0.05 to 0.49)	61.1 (0.39 to 0.84)	22.2 (−0.05 to 0.49)	61.1 (0.39 to 0.84)	48.1 (0.29 to 0.67)

The numbers in parentheses represent 95% confidence interval; NSCLC, non-small cell lung cancer; PPV, positive predictive value; and NPV, negative predictive value.

The average SUVmax for visually positive regions was 3.0 ± 1.2 with 4DST and 3.1 ± 0.9 with FDG, with no significant difference (*P* = 0.40). There also was no significant difference between the SUVmax for true positives and that for false positives with 4DST (2.6 ± 0.9 vs. 3.2 ± 1.3; *P* = 0.12) and with FDG (3.5 ± 0.8 vs. 2.9 ± 1.0; *P* = 0.18). The ascending aorta SUVmax was lower with 4DST (0.7 ± 0.2) than with FDG (1.9 ± 0.3; *P* < 0.001). The L/B ratio was significantly higher with 4DST (5.0 ± 2.9) than with FDG (1.7 ± 0.5; *P* < 0.001). No significant difference was observed between true positives and false positives with 4DST (4.1 ± 2.2 vs. 5.4 ± 3.2; *P* = 0.16), but there was a significant difference between them with FDG (2.1 ± 0.6 vs. 1.5 ± 0.4; *P* = 0.03).

The results for staging are shown in Table 3. The nodal stage based on 4DST-PET/CT imaging was overstaged in 16 patients (59.3%), with no understaged cases, whereas the stage based on FDG-PET/CT imaging was overstaged in nine patients (33.3%) and understaged in five patients (18.5%). Both CECT and FDG imaging showed no evidence of distant metastatic lesions in all patients included in the study. For two false positives by all three imaging modalities (4DST, FDG, and CECT), one was histopathologically proven to be reactivation of sarcoidosis, and the other was proven to be chronic inflammation.

**Table 3:** Comparison of nodal staging by 4DST and FDG with pathological results

**Nodal staging**	**PET/CT**	
	**4DST**	**FDG**
Over diagnosis	16	9
Correct	11	13
Underestimate	0	5

### Spread lesion and recurrence after PET studies

At the time of analysis, all patients were alive, but eight patients had cancer recurrences as LN metastasis (*n* = 2), pleural dissemination (*n* = 2), brain metastasis (*n* = 2), pulmonary metastasis (*n* = 1), or as a bone metastasis (*n* = 1). Table 4 shows the results for spread lesion (*n* = 4) or recurrence (*n* = 19) for 23 patients according to the nodal staging based on 4DST and FDG imaging. Cases with 4DST uptake in the nodal lesion showed a high frequency of spread lesion or recurrence, compared to the results of FDG. The DFS rate with positive 4DST uptake in nodal lesions was 0.35 (95% CI 0.13 to 0.58), which was lower than the 0.83 rate (95% CI 0.54 to 1.13) with negative findings (*P* = 0.04), whereas the DFS rate with positive FDG uptake in nodal lesions was 0.56 (95% CI 0.23 to 0.88), which was higher than the 0.50 (95% CI 0.24 to 0.76) with negative findings (*P* = 0.79). The recurrence-free rate for 2 years with positive 4DST uptake in nodal lesions was 0.46 (95% CI 0.19 to 0.73), which was lower than the 0.83 rate (95% CI 0.53 to 1.13) with negative findings (*P* = 0.12). The recurrence-free rate with positive FDG uptake in nodal lesions was 0.43(95% CI 0.06 to 0.80), which was only slightly lower than the 0.59 rate (95% CI 0.30 to 0.86) with negative findings (*P* = 0.96).

**Table 4:** The number of patients according to nodal staging and spread lesion or recurrence

**Nodal staging**	**Clinical and pathological TNM classification**	**Preoperative PET/CT**	
		**4DST**	**FDG**
N3	0	3 (3)	1 (1)
N2	7 (7)	11 (6)	5 (3)
N1	3 (1)	3 (2)	3 (1)
N0	13 (4)	6 (1)	14 (7)

The numbers in parentheses represent the case with spread lesion or recurrence within two years.

Logistic regression analysis of potential prognostic factors for the presence of spread lesion or recurrence over 2 years showed that 4DST was the most influential factor (odds ratio 29.08), but it was not significant (*P* = 0.05). Lung lesion 4DST was the second most influential factor (odds ratio 8.39; Table 5).

**Table 5:** Logistic regression analysis of potential prognostic factors for the presence of spread lesion or recurrence over two years

**Variables**	**Univariate analysis**			**Multivariate analysis**		
	**Odds ratio**	**95% CI**	***P *** **value**	**Odds ratio**	**95% CI**	***P *** **value**
Nodal staging by 4DST (N1 to 3 vs N0)	9.17	0.86 to 97.67	0.07	29.08	0.95 to 894.08	0.05
Nodal staging by FDG (N1 to 3 vs N0)	1.25	0.23 to 6.71	0.79	0.63	0.05 to 8.24	0.72
4DST uptake (SUVmax) in lung tumor (>2.4 vs ≤2.4)	6.29	0.58 to 68.42	0.13	8.39	0.20 to 348.94	0.26
FDG uptake (SUVmax) in lung tumor (>3 vs ≤3)	2.40	0.44 to 12.98	0.31	3.23	0.07 to 150.84	0.55
Lung tumor diameter (>3 cm vs ≤3 cm)	1.17	0.22 to 6.08	0.85	0.16	0.01 to 4.87	0.30
Age (>60 years vs ≤60 years)	0.75	0.13 to 4.49	0.75	0.42	0.02 to 9.70	0.59

CI, confidence interval.

## Discussion

The present study demonstrated that 4DST has a better sensitivity for N staging than FDG in NSCLC, both on a per-patient basis and on a per-node basis. In addition, 4DST uptake in a nodal lesion may predict the spread lesion and recurrence more sensitively than other factors.

The biggest difference between 4DST and FDG imaging was the background uptake in the mediastinum. Since positive uptake was not different between 4DST and FDG, 4DST could show accumulation in LNs more easily because of greater contrast to the ‘background’. The same facts are also reflected in the difference in the L/B ratio between 4DST and FDG. However, it was difficult to set a cutoff value for 4DST uptake to distinguish true positives from false positives because the true- and false-positive 4DST uptakes overlapped. That was a limitation of 4DST for evaluating nodal staging.

As mentioned previously, FDG PET has shown higher sensitivity and specificity than CT in detecting LN metastasis in lung cancer [5,28]. For small LNs with a diameter less than 1 cm, FDG PET also has higher sensitivity than CT for detecting regional LN metastasis [29]. FDG-PET/CT showed variable sensitivity, but consistently high specificity [6]. However, the sensitivity of FDG-PET/CT decreased to 42% in stage T1 NSCLS [8]. Stiles et al. reported that lymph node metastasis was pathologically detected in 11.7% of patients with clinical stage IA lung cancer [30]. These facts indicate a limitation of FDG PET/CT. The present study showed lower sensitivity and specificity for FDG PET/CT than has been reported previously [5,28]. The likely causes are (1) the evaluation of small LN groups that would be treated as negative by CT imaging but could be detected as positive by the surgical procedure in the present study, (2) the inclusion of many T1-stage cases (41% of all cases included in the present study), and (3) the inclusion of many cases with micrometastases in lymph nodes. In fact, when the diameter of lymph node with 10 mm or greater in short axis was regarded as positive, the diagnostic result from CECT by two board-certified radiologists was lower (sensitivity 29.4% [5/17], specificity 89.6% [95/106] per nodal station) than previously reported [5,28].

The use of 3′-deoxy-3′-^18^F-fluorothymidine (FLT) for cellular proliferation imaging has yielded good clinical results [31]. In contrast, the diagnostic performance of FLT for staging and restaging of thoracic tumors has been reported to be inadequate [32,33]. Several reports have shown that FLT has better specificity and PPV than FDG, but lower sensitivity [32-35]. FLT has also shown a limited ability in discriminating reactive and metastatic LNs in head and neck cancers [36]; this is caused by the FLT uptake to reactive B-lymphocyte proliferation [37]. Chronic inflammatory granulomatous lesions include more than a small Ki-67-positive lymphocyte fraction [38]; therefore, increased FLT uptake was confirmed in granulomas that arose after radiation and chemotherapy [35]. FLT is not incorporated into DNA because of the lack of a 3′-hydroxyl, unlike thymidine [39]. On the other hand, considering that 4DST is incorporated into DNA [24,25], 4DST is expected to discriminate metastatic LNs from others, but it also has a possibility of accumulating with reactive proliferation. According to an animal study, very low levels of 4DST uptake were observed in subacute inflammatory areas, which reflects the cell proliferation status of inflammatory tissues [40]. In fact, the present results showed no significant difference in 4DST uptake between true positives and false positives. Although 4DST is a proliferation marker, it accumulated not only in malignant lesions, but it also reacted to chronic inflammatory lesions; these facts were also reflected by 4DST uptake in sarcoid lesions. Furthermore, the low PPV and overstaging of 4DST for LN lesions might be partially due to reactive LNs. Low specificity is one of the limitations of 4DST for nodal staging in lung cancer.

One focal point of the present study was the relationship between clinical nodal staging by PET and prognosis. Cases with positive findings on 4DST in the nodal area showed a high incidence of recurrence and lesion extension compared to FDG. Except for the case with a histologically proven sarcoid reaction, positive 4DST uptakes were present along lymphatic channels from the primary lesion so that they seemed to be valid for suspecting metastatic LN lesions. They might be stimulated by the primary lesion and indicate the spread of lesion, even though no malignancy was proven.

The high sensitivity of 4DST for nodal metastasis indicates that it may be detecting micro LN metastasis. On the other hand, the low PPV was a disappointing result with respect to providing accurate LN staging, but positive 4DST uptake in LNs appeared to be a strong factor for predicting the spread lesion or a high possibility of early recurrence. If the false-negative 4DST uptake was regarded as one of the reactive features from the surroundings, it might be caused by inflammatory mediators such as chemokines, cytokines, and prostaglandins associated with cancer-related inflammation [41,42]. Nodal staging with 4DST and uptake in the primary lung tumor of 4DST were more influential prognostic factors than the same features with FDG, but further evaluation is needed.

A limitation for M staging with 4DST was anticipated due to physiological uptake in the liver, kidney, and bone marrow. Since only a small number of patients was examined, larger patient samples need to be examined to verify the diagnostic accuracy of 4DST PET for N staging and its potential for predicting prognosis.

## Conclusion

4DST PET/CT is sensitive for detecting mediastinal lymph node metastasis in NSCLC, but its low specificity is a limitation. However, it may be helpful in predicting the prognosis of NSCLC.

## Abbreviations

4DST: 4′-[methyl-^11^C]-thiothymidine; AJCC: American Joint Committee on Cancer; CE: contrast-enhanced; CI: confidence intervals; CT: computed tomography; DFS: disease-free survival; EBUS-TBNA: endobronchial ultrasonography-transbronchial needle aspiration; FDG: 2-deoxy-2-^18^F-fluoro-d-glucose for detecting regional; FLT: 3′-deoxy-3′-^18^F-fluorothymidine; L/B: lesion-to-background; LN: lymph node; MRI: magnetic resonance imaging; N1G: N1 group; NPV: negative predictive value; NSCLC: Non-small cell lung cancer; PET: Positron emission tomography; PPV: positive predictive value; ROI: regions of interest; SUVmax: maximum standardized uptake value.

## Competing interests

The authors declare that they have no competing interests.

## Authors' contributions

RM and KK are guarantors of integrity of the entire study. RM, JT, KI, KK are responsible for the study concepts and design. RM, JT, KI, KI, and KK participated in the literature research. RM, HI, AS, KN, KK, and MO performed the clinical studies. RM, YM, MM, MO, and KK performed the data analysis/interpretation. RM, JT, KI, KI, and KK did the manuscript preparation. RM, JT, KI, KI, and KK participated in the manuscript revision/review. All authors read and approved the final manuscript.
